# Genicular artery embolization in a patient with popliteal artery agenesis: a case report

**DOI:** 10.1186/s42155-025-00631-1

**Published:** 2025-12-13

**Authors:** Paula Krüselmann, Nicolas Steinfort, Arian Taheri Amin, Peter Minko

**Affiliations:** Department of Diagnostic and Interventional Radiology, University Hospital Düsseldorf, Medical Faculty, Düsseldorf, Germany

**Keywords:** Genicular artery embolization, Popliteal artery agenesis, Vascular anomaly, Knee osteoarthritis, Endovascular therapy, Collateral circulation

## Abstract

**Background:**

Genicular artery embolization (GAE) is an emerging, minimally invasive therapy for refractory knee osteoarthritis (OA), targeting pathological synovial hypervascularization. While technically well established in typical anatomy, rare congenital anomalies such as popliteal artery (PA) agenesis present unique procedural challenges and demand careful adaptation of endovascular technique.

**Case presentation:**

A 56-year-old woman with refractory right knee OA and polymyalgia rheumatica presented with persistent pain despite extensive medical and surgical therapies. Angiography revealed complete absence of the PA, with distal lower limb perfusion entirely maintained through a dense network of arterial anastomoses. Detailed angiographic assessment and superselective catheterization allowed targeted embolization of hypervascular synovial branches while preserving critical collaterals. The procedure was technically successful and uneventful, providing substantial pain relief within two weeks, maintained at 3, 6, and 12 months. At 18 months, symptoms recurred and repeat GAE was considered.

However, the symptoms resolved spontaneously, and no further embolization was required. Only mild swelling on exertion persisted. At the two-year follow-up, the patient reported sustained pain relief without further interventions.

**Conclusions:**

This case illustrates that GAE can be safely and effectively performed even in the presence of rare congenital vascular anomalies such as PA agenesis. Meticulous angiographic assessment, precise differentiation of synovial from distal perfusion territories, and a tailored embolization strategy are essential to achieve safe and durable outcomes in such anatomically challenging scenarios. This case report underscores the adaptability of endovascular techniques and expands the evidence base for GAE in patients with rare vascular variants.

## Background

Genicular artery embolization (GAE) has emerged as a promising non-surgical intervention for patients suffering from chronic knee pain due to osteoarthritis (OA), particularly when conservative treatments fail or surgery is not an option [1, 2]. By targeting pathological neovascularization within the genicular arteries (GAs), GAE has demonstrated safety and efficacy in multiple meta-analyses [3–7].

The target vessels of GAE typically originate from the superficial femoral artery (SFA) and the popliteal artery (PA). Anatomical variants of the PA are not uncommon: high division, hypoplastic or aplastic branches and anomalous branching patterns have been reported in up to 3–5% of the population [8]. In contrast, complete congenital agenesis of the popliteal artery is exceptionally rare, with only two cases described in the literature to date [9, 10].

Embryologically, the popliteal artery arises from the axial artery of the lower limb, which initially develops as a branch of the internal iliac artery and runs along the posterior aspect of the limb bud as the so-called sciatic artery [11]. By the sixth to eighth week of gestation, the external iliac artery elongates to form the femoral artery, which establishes anastomoses with the distal axial artery [12]. As the femoral system becomes dominant, the proximal sciatic artery regresses, while its midportion persists as the definitive PA and its distal segment contributes to the peroneal artery [8]. Failure of this sequence of remodeling and regression can result in hypoplasia or complete agenesis of the PA. Disturbances in key vascular signaling pathways, such as VEGF and Notch, which are essential for angiogenesis and arterial specification, have been proposed as contributing factors [13]. Premature involution of the axial artery before the femoral system is fully established may also lead to agenesis [14]. In such cases, hypertrophied genicular and collateral arteries maintain perfusion to both: the knee and the distal extremity. Compared to more frequent congenital variants of the popliteal artery, such as high division or hypoplasia, which have been reported in up to 3–5% of the population [6, 8], complete congenital agenesis remains exceptionally rare, with only a few cases described to date [9, 10]. Such vascular anomalies present unique challenges during endovascular procedures, as they fundamentally alter the collateral circulation and increase the risk of non-target embolization.

As the number of GAE procedures continues to rise, the detection of anatomical variants such as PA agenesia is likely to become more frequent. The absence of the PA places a greater functional load on genicular anastomoses, making precise imaging, superselective catheterization, and a tailored embolization strategy essential to avoid ischemic complications while achieving therapeutic success. The technical considerations and clinical outcome in this rare setting provide valuable insights for IRs performing GAE in patients with unexpected vascular variants.

## Case presentation

### Patient history

A 56-year-old woman with a long-standing history of bilateral knee OA, further complicated by polymyalgia rheumatica, presented with persistent medial right knee pain. Her symptoms had been refractory to conservative treatments, including NSAIDs, corticosteroids, opioids, and multiple intra-articular injections. Despite having undergone arthroscopic partial meniscectomy and synovectomy, her symptoms persisted. She declined total knee arthroplasty and was referred for GAE.

### Procedure

**Fig. 1:**
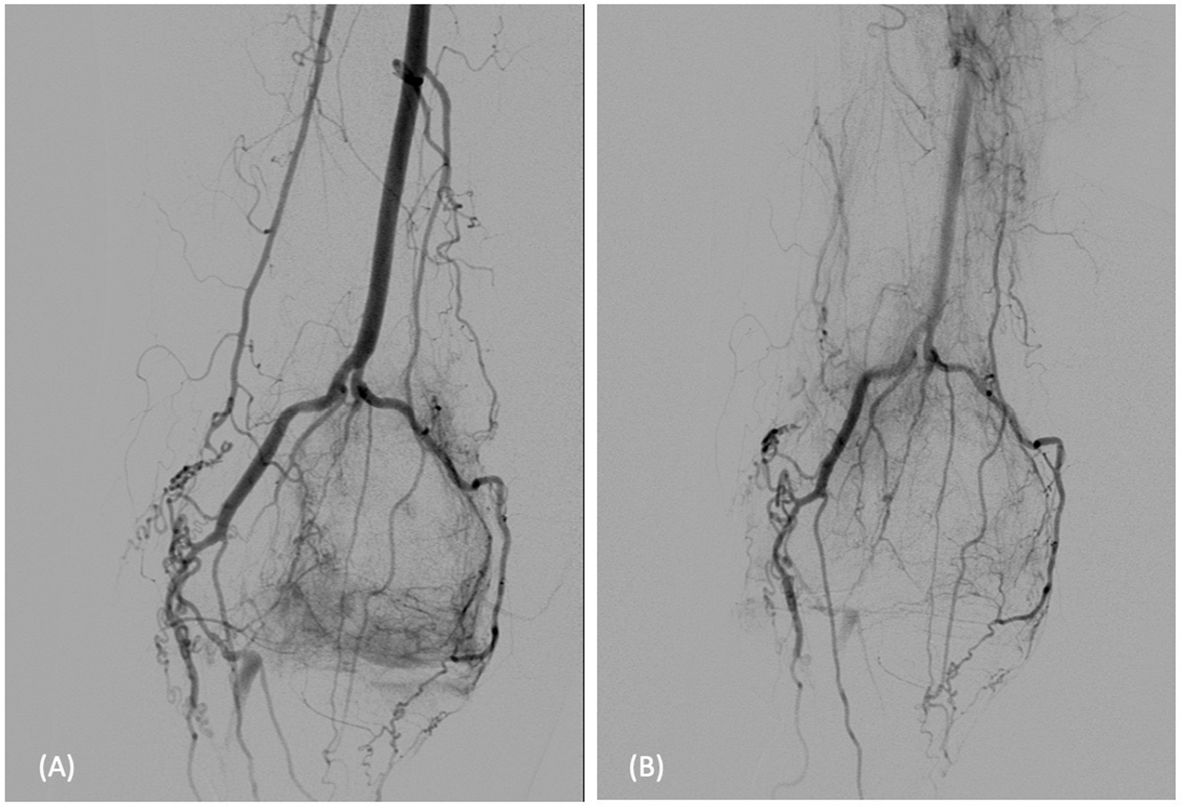
Pre and Post overview. **A** Pre-interventional digital subtraction angiography (DSA) of the distal superficial femoral artery confirming *complete congenital absence* of the popliteal artery. Distal perfusion of the lower limb is maintained entirely through hypertrophied genicular collaterals, without any evidence of thrombotic material or arterial wall irregularities, supporting a congenital etiology. **B** Post-interventional overview DSA from a similar position demonstrates a reduction in synovial blush while hypertrophied crural collaterals remain patent

The procedure was performed under local anesthesia following ultrasound-guided antegrade puncture of the right common femoral artery without the use of an introducer sheath to minimize vascular access size. Initial DSA was conducted at the mid-third of the SFA via a 4 F Cobra catheter (Infiniti®, Cordis Medical, Austria) using iodinated contrast medium (300 mg/mL, Accupaque®, GE HealthCare, USA). DSA revealed complete absence of the P2 segment of the PA (Fig. 1A) [9]. Instead, distal perfusion of the P3 segment and below-knee vessels was maintained entirely via enlarged collateral genicular branches. The angiographic appearance, combined with the absence of calcifications or thrombus, confirmed a congenital anomaly rather than acquired vessel occlusion.

**Fig. 2:**
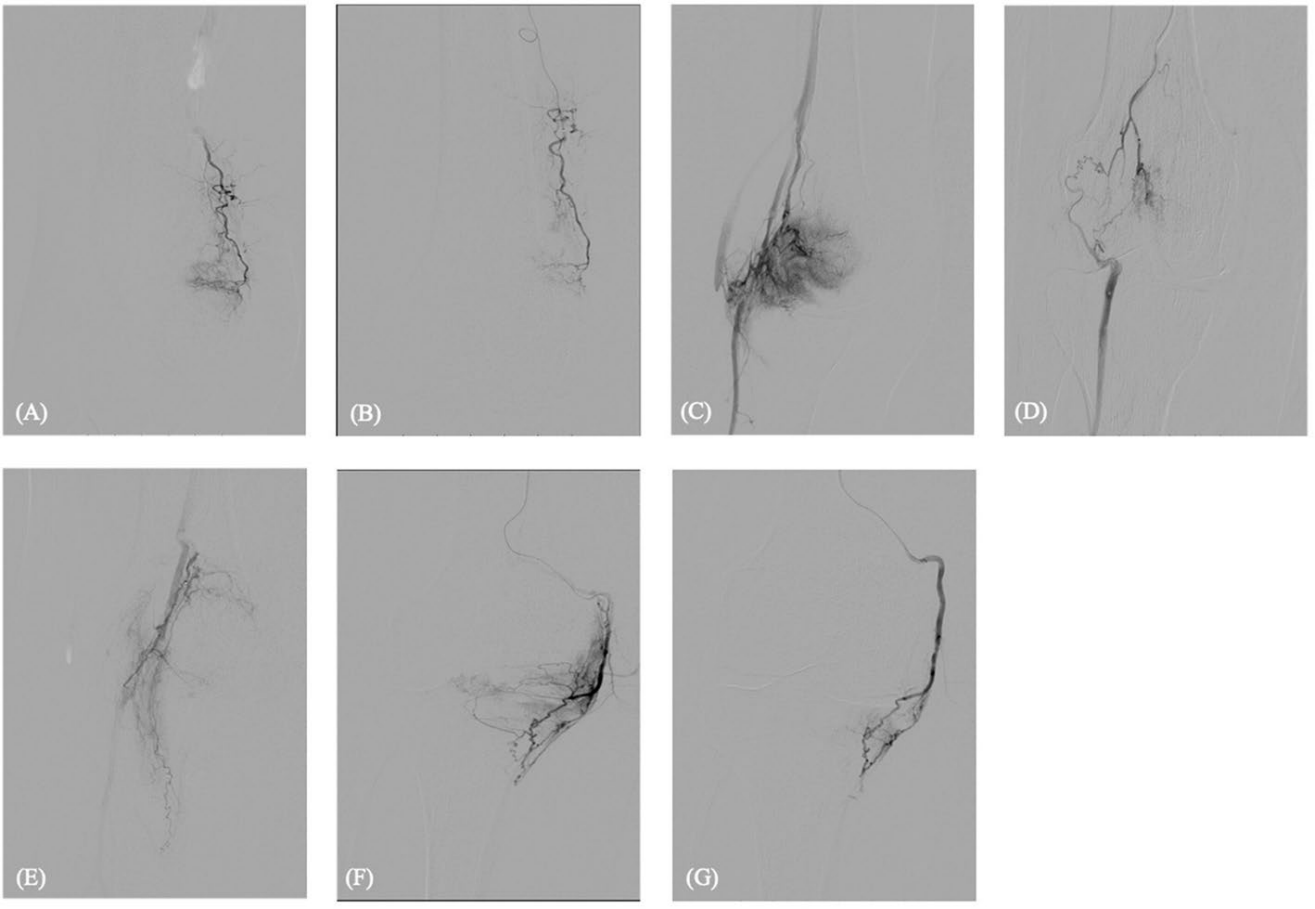
Superselective angiography of genicular branches. **A** Pre-embolization imaging of the descending genicular artery shows a dense synovial hypervascular blush. Following embolization with 0.8 ml of a mixture of permanent embolic material (100–300 μm Embospheres®, Merit Medical, USA) diluted in 10 mL of iodinated contrast agent, a marked reduction in vascular blush is observed, with preservation of the parent vessel (**B**). **C** Pre-embolization imaging of the inferior medial genicular artery reveals prominent synovial hypervascular blush. After embolization with 0.8 ml of the mixture mentioned above, a clear reduction in blush intensity is seen, with the parent vessel remaining intact (**D**). **E** Superselective angiography of a lateral genicular branch revealed dense collaterals supplying the distal crural arteries. To avoid the risk of lower limb ischemia, embolization was withheld. **F** The inferomedial genicular artery showed moderate hypervascularity without evoked pain. Due to extensive distal crural anastomoses, embolization was cautiously performed with 0.3 ml of the mixture mentioned above, resulting in reduced blush and preserved flow (**G**)

Superselective catheterization of medial and lateral genicular branches was performed using a 1.7F microcatheter (Pursue®, Merit Medical, USA), with particular attention to anastomoses between genicular arteries and distal crural arteries. The descending genicular artery demonstrated marked hypervascularity, and superselective angiography reproduced the patient’s pain (Fig. 2A). Identification of the target areas was guided by dynamic DSA findings in combination with clinical correlation. During superselective angiography, reproduction of the patient’s characteristic pain pattern was used to confirm symptomatic vascular territories, as described in previous GAE studies [15, 16]. This functional correlation, together with visualization of hypervascular synovial blush, defined the target zones for embolization.

Embolization was performed using 0.8 ml of a mixture of permanent embolic material (100–300 μm Embospheres®, Merit Medical, USA) diluted in 10 mL of the iodinated contrast agent mentioned above. The superomedial genicular artery showed a similar hypervascular blush with evoked pain, embolized with 0.8 ml of the mixture mentioned above (Fig. 2B). The inferomedial genicular artery exhibited moderate hypervascularity without evoked pain while extensive anastomoses with the distal crural arteries were seen. Thus, embolization was performed cautiously using a reduced volume of 0.3 ml (Fig. 2C). Embolic material was injected in small aliquots to selectively “prune” abnormal neovessels while preserving the parent artery’s flow. An unnamed lateral branch that provided critical supply to the crural arteries was intentionally spared to avoid compromising distal perfusion (Fig. 2D). A final DSA series from the distal SFA confirmed patency of the lower leg arteries (Fig. 1B). To minimize non-target embolization and post-procedural skin discoloration, ice packs were applied around the knee joint during the procedure.

Vascular and non-vascular complications were assessed with duplex ultrasound and clinical evaluation four hours after the procedure upon discharge and 24 h post-procedure. No complications were reported.

The patient experienced substantial pain relief within two weeks. Follow-up visits at three, six and twelve months showed continued improvement. After 18 months, the patient experienced a slight return of pain, mainly in association with exertional swelling, which prompted consideration of repeat GAE. As the symptoms subsided spontaneously, re-embolization was not required. Only mild exertional swelling remained. At the 2.5-year follow-up, the patient continued to report stable pain relief without the need for additional treatment.

## Conclusion

This case demonstrates the central role of detailed vascular imaging and a thoughtful embolization strategy when performing GAE, particularly in the context of rare vascular anomalies. The absence of the PA places a greater functional load on anastomoses. As a result, the IR faces the technically challenging dilemma of adequately embolizing the pathological neovascularization while minimizing the risk of non-target embolization via the excessive anastomotic network, which could lead to acute limb ischemia.

PA agenesis results from embryological failure of axial artery development, leading to highly atypical vascular patterns [4]. Differentiating a congenital absence from an acquired arterial occlusion is critical. In this patient, the absence of arterial wall calcification or thrombotic material, along with the presence of robust, well-established collateral networks, strongly supported a congenital etiology [7].

Performing GAE in patients with vascular anomalies like this one requires careful attention to diagnostic angiography. Superselective catheterization is mandatory to preserve critical collateral pathways while achieving therapeutic embolization. While DSA offers real-time procedural guidance and is considered the gold standard for evaluation of vascular anatomy, pre-procedural CTA or MRA would have improved procedural planning in this case [17]. However, these imaging modalities are not routinely included in the pre-procedural workup for GAE, and given the low incidence of vascular anomalies, their integration into the standard diagnostic algorithm appears inefficient. As a cost-effective and low-effort alternative, duplex ultrasound appears to be a reasonable screening tool for evaluating lower limb vessels prior to GAE. In cases of suspicious findings, additional imaging with CTA or MRA may be warranted.

In this patient, recurrence of symptoms occurred at 18 month despite the durability of GAE demonstrated in the GENESIS study, where most patients experienced sustained pain relief beyond 24 month [18]. However, this recurrence was transient and resolved spontaneously without the need for repeat embolization, resulting in a sustained pain-free status at the 2.5-year follow-up. This earlier recurrence characterized by mild pain but predominantly swelling after exertion, may be explained by the unusually pronounced collateral network associated with the popliteal artery agenesis. As Taheri et al. have demonstrated, extensive anastomoses can maintain hyperperfusion in a vascular territory retrogradely, even after the antegrade embolization [19]. In this case, the exceptional vascular anatomy, with numerous collateral pathways and anastomoses supplying both, the knee and the lower leg, likely maintained synovial hypervascularization via untreated territories and contributed to the earlier symptom recurrence, despite technically successful embolization [20]. Furthermore, not all pathological vessels were embolized due to the atypical anatomy and critical anastomoses supplying the lower leg. Evidence suggests that, in event of recurrence, repeat GAE is both feasible and effective and regular clinical follow-up remains essential for long-term patient management [3].

In the presence of popliteal artery agenesis, the hemodynamic situation differs substantially, as distal limb perfusion relies entirely on genicular and collateral pathways. This configuration increases the risk of non-target embolization and requires meticulous assessment of collateral flow before and during the procedure. In our case, superselective catheterization and cautious, stepwise embolization ensured preservation of distal perfusion while achieving adequate synovial devascularization.

GAE can be performed safely and successfully even in patients with complex congenital vascular anomalies such as popliteal artery agenesis. A successful outcome hinges on meticulous vascular assessment and a carefully tailored embolization strategy that respects the dual role of genicular arteries in both mediating synovitis and supplying critical joint structures. This case further underscores the importance of interventional adaptability and precise imaging in ensuring safe and effective treatment outcomes.

## Data Availability

All data generated or analysed during this study are included in this published article.

## References

[1] Bagla S, Piechowiak R, Sajan A, et al. Genicular artery embolization for the treatment of knee osteoarthritis: results from a prospective multicenter trial. Cardiovasc Intervent Radiol. 2022;45(10):1552–60.

[2] Neville RF Jr, Franco CD, Anderson RJ, et al. Popliteal artery agenesis: a new anatomic variant. J Vasc Surg. 1990;12(5):573–6. 2231969 10.1067/mva.1990.24156

[3] Landers J, Tins B, Cool P, Hayton MJ, Satchithananda K, Rajani R, et al. Genicular artery embolisation in patients with osteoarthritis of the knee (GENESIS) trial: a randomised, double-blind, sham-controlled trial. Bone Jt Open. 2023;4(2):94–102.

[4] Senior HD. The development of the arteries of the human lower extremity. Am J Anat. 1919;25(1):54–95.

[5] Little MW, Gibson M, Briggs J, et al. Genicular artery embolization in patients with osteoarthritis: a systematic review and meta-analysis. Eur Radiol. 2023;33(1):25–36.

[6] Kim D, Orron DE, Skillman JJ. Surgical significance of popliteal arterial variants: a unified angiographic classification. Ann Surg. 1989;210(5):776–81. 2589890 10.1097/00000658-198912000-00014 PMC1357871

[7] Park JC, Jungs GS, Suh KK. Popliteal artery agenesis detected by CT angiography: a rare vascular anomaly. J Korean Soc Radiol. 2018;79(4):233–6.

[8] Adams RH, Alitalo K. Molecular regulation of angiogenesis and lymphangiogenesis. Nat Rev Mol Cell Biol. 2007;8(6):464–78. 17522591 10.1038/nrm2183

[9] Wilms LM, Jannusch K, Weiss D, Steinfort N, Ziayee F, Antoch G, et al. Transarterial microembolization for the management of refractory chronic joint pain in osteoarthritis. Rofo. 2024. 10.1055/a-2288-5743. 38740066 10.1055/a-2288-5743

[10] Korchi AM, Cengarle-Samak A, Okuno Y, Martel-Pelletier J, Pelletier JP, Boesen M, et al. Inflammation and hypervascularization in a large animal model of knee osteoarthritis: imaging with pathohistologic correlation. J Vasc Interv Radiol. 2019;30:1116–27. 30935868 10.1016/j.jvir.2018.09.031

[11] Tomanek RJ, Hansen-Smith FM. Development of the arterial system. In: The Cardiovascular System. Vol 1. 2nd ed. New York: Raven Press; 1991:137–212.

[12] Wang Y, Nakayama M, Pitulescu ME, et al. Ephrin-B2 controls VEGF-induced angiogenesis and lymphangiogenesis. Nature. 2010;465(7297):483–6. 20445537 10.1038/nature09002

[13] Wilting J, Müller-Hülsbeck S, Becker J. Embryology of the vascular system and pathogenesis of vascular malformations. Vasa. 2014;43(4):219–30.

[14] Dias-Neto M, Casella IB, Yamane Y, De Luccia N. Comparison of digital subtraction angiography and computed tomography angiography in the evaluation of peripheral arterial disease. Clin (Sao Paulo). 2018;73:e324.

[15] Wang B, Liang KW, Chen CH, Wang CK. Transcatheter Arterial Embolization for Alleviating Chronic Musculoskeletal Pain and Improving Physical Function: A Narrative Review. Diagnostics (Basel). 2022;13(1):134. 36611426 10.3390/diagnostics13010134 PMC9818587

[16] Taheri Amin A, Frommhold I, Huebner A, Boschheidgen M, Frenken M, Jannusch K, et al. Genicular artery embolization in moderate to severe knee osteoarthritis: technique, safety and clinical outcome. Cardiovasc Intervent Radiol. 2025;48(3):340–50. 39971795 10.1007/s00270-025-03983-2 PMC11889044

[17] Epelboym Y, Mandell JC, Collins JE, Burch E, Shiang T, Killoran T, et al. Genicular artery embolization as a treatment for osteoarthritis-related knee pain: a systematic review and meta-analysis. Cardiovasc Intervent Radiol. 2023;46:760–9. 36991094 10.1007/s00270-023-03422-0

[18] Little MW, Tins B, Cool P, Hayton MJ, Satchithananda K, Rajani R, et al. Genicular artery embolisation in patients with osteoarthritis of the knee (GENESIS): a randomised, double-blind, sham-controlled trial. Bone Jt Open. 2023;4(2):94–102.

[19] Amin AT, Ziayee F, Boschheidgen M, Hübner A, Kemmer E, Weiss D, Wilms LM, Tietz E, Jannusch K, Minko P. Road to genicular artery embolization: importance of the anastomotic network. Cardiovasc Intervent Radiol. 2025 Jul 24. 10.1007/s00270-025-04121-8. Epub ahead of print. PMID: 40707840. 10.1007/s00270-025-04121-8 PMC12665632 40707840

[20] Poursalehian M, Bhia I, Ayati Firoozabadi M, Mortazavi SMJ. Genicular artery embolization for knee osteoarthritis: a comprehensive review. JBJS Rev. 2023;11:e23. 10.2106/JBJS.RVW.23.00082 37683080

